# Elevated plasma Galectin-3 is associated with major adverse kidney events and death after ICU admission

**DOI:** 10.1186/s13054-021-03878-x

**Published:** 2022-01-06

**Authors:** L. Boutin, M. Legrand, M. Sadoune, A. Mebazaa, E. Gayat, C. E. Chadjichristos, F. Dépret

**Affiliations:** 1 Department of Anaesthesiology, Critical Care Medicine and Burn Unit, AP-HP, Saint-Louis Hospital, DMU Parabol, FHU PROMICE, Université de Paris, Paris, 75010 France; 2 INSERM, UMR 942, MASCOT: Cardiovascular Marker in Stress Condition, Lariboisière Hospital, Université de Paris, Paris, 75010 France; 3 Department of Anesthesiology and Peri-Operative Medicine, Division of Critical Care Medicine, University of California – UCSF Medical Center, 500 Parnassus Ave, San Francisco, CA 94143 USA; 4 INSERM, UMR 1155, CORAKID, Tenon Hospital, Université de Paris, 75020 Paris, France

**Keywords:** Acute kidney injury, Galectin-3, Major Adverse Kidney Event, Renal biomarker

## Abstract

**Background:**

Galectin-3 (Gal-3) is a proinflammatory and profibrotic protein especially overexpressed after Acute Kidney Injury (AKI). The early renal prognostic value of Gal-3 after AKI in critically ill patients remains unexplored. The objective was to evaluate the prognostic value of plasma level of Gal-3 for Major Adverse Kidney Events (MAKE) and mortality 30 days after ICU admission across AKI stages.

**Methods:**

This is an ancillary study of a prospective, observational, multicenter cohort (FROG-ICU). AKI was defined using KDIGO definition.

**Results:**

Two thousand and seventy-six patients had a Gal-3 plasma level measurement at ICU admission. Seven hundred and twenty-three (34.8%) were females and the median age was 63 [51, 74] years. Eight hundred and seven (38.9%) patients developed MAKE, 774 (37.3%) had AKI and mortality rate at 30 days was 22.4% (*N* = 465). Patients who developed MAKE had higher Gal-3 level at admission compared to patients without (30.2 [20.8, 49.2] ng/ml versus 16.9 [12.7, 24.3] ng/ml, *p* < 0.001, respectively. The area under the receiver operating characteristic curve of Gal-3 to predict MAKE was 0.76 CI_95%_ [0.74–0.78], *p* < 0.001. Gal-3 was associated with MAKE (OR 1.80 CI_95%_ [1.68–1.93], *p* < 0.001, non-adjusted and OR 1.37 CI_95%_ [1.27–1.49], *p* < 0.001, adjusted). The use of Gal-3 improved prediction performance of prediction model including SAPSII, Screat_adm_, pNGAL with a NRI of 0.27 CI_95%_(0.16–0.38), p < 0.001. Median Gal-3 was higher in non-survivors than in survivors at 30 days (29.2 [20.2, 49.2] ng/ml versus 18.8 [13.3, 29.2] ng/ml, *p* < 0.001, respectively).

**Conclusion:**

Plasma levels of Gal-3 were strongly associated with renal function, with an increased risk of MAKE and death after ICU admission.

*Trial registration* ClinicalTrials.gov NCT01367093. Registered on 6 June 2011.

**Graphical abstract:**

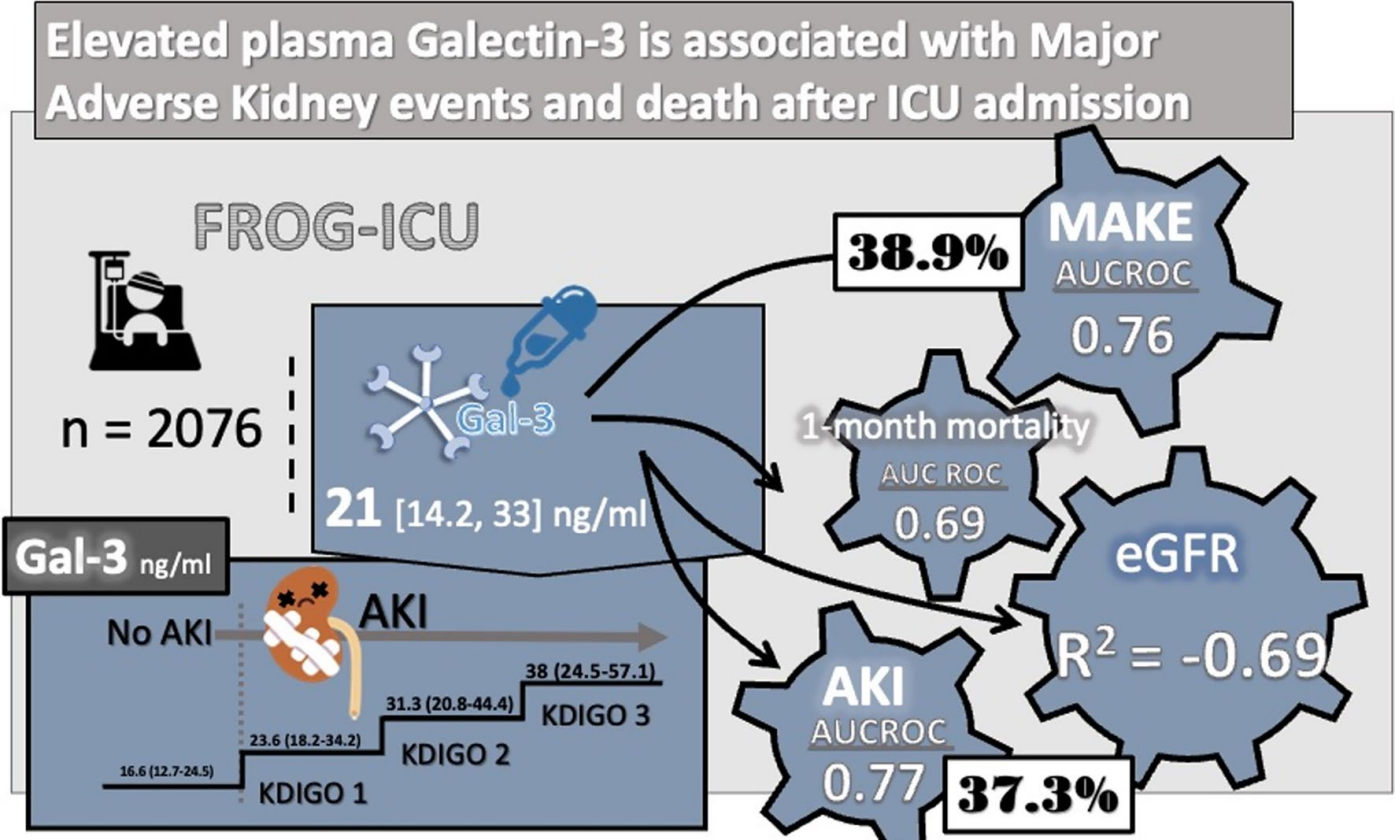

**Supplementary Information:**

The online version contains supplementary material available at 10.1186/s13054-021-03878-x.

## Background

Acute kidney injury (AKI) is a frequent complication of critical illnesses and has been repeatedly associated with mortality and cardiovascular events [1, 2]. The mechanisms linking AKI to poor outcomes are not fully understood yet. Galectin-3 (Gal-3) is a 30kD lectin protein, ubiquitously expressed in the human body [3]. Gal-3 is mostly produced by macrophages and promotes inflammation and post-injury fibrosis [4, 5]. Experimental studies showed that Gal-3 leads to both kidney and cardiac injury and fibrosis [6–8] which is prevented by pharmacological or genetic inhibition [8, 9]. In addition, in a preclinical model, AKI led to an overexpression of Gal-3 and further cardiac damage, inflammation and fibrosis [8]. More recently, in a model of sepsis (i.e., caecal ligature and puncture), Gal-3 was associated with the risk of AKI and its pharmacological blockage using modified citrus pectin prevented sepsis-associated AKI [9]. These translational data suggest that Gal-3 could play a role both in the development of renal damage and remote organs damage after AKI. However, the association between admission plasmatic Gal-3 level and outcomes in critically ill patients has never been explored. Therefore, the objectives of this study were to evaluate the association between plasma Gal-3 level at intensive care unit (ICU) admission with Major Adverse Kidney Events (MAKE), and mortality at day 30.

## Methods

### Study design

This study is an ancillary study of the French and euRopean Outcome reGistry in Intensive Care Units (FROG-ICU) cohort study (ClinicalTrials.gov: NCT01367093). FROG-ICU was a European prospective, observational, and multinational study in twenty-four centers from five countries (France, Belgium, The Netherlands, Italy, and Germany), including patients who received mechanical ventilation and/or vasopressors or inotropes. The protocol was previously described elsewhere [10]. Patients have been recruited from August 2011 to June 2013.

### Collection of patient data

Upon admission, demographics (i.e., age, gender), body mass index, presence of sepsis or septic shock, type of ICU admission, organ dysfunction and severity scores (Sequential Organ Failure Assessment [SOFA] [11], Simplified Acute physiology score [SAPS II] [12]), pre-existing comorbidities treated within the past year, past medical history, laboratory values, admission urine output, as well as organ support were recorded. During the first week after patient enrollment, the following data were assessed and/or collected daily: SOFA score, ventilation status, Glasgow Coma Scale, need for renal replacement therapy (RRT) and vasopressor treatment. On the third month, discharge status or mortality was recorded in all study participants.

### Endpoint definition

AKI definition was based on the Kidney Disease Global Outcome (KDIGO) definition using serum creatinine (Screat) during the first 7 days after admission [13].

MAKE was defined as a composite outcome of the following criteria, 30 days after admission or at ICU discharge, whatever came first: death within 30 days and/or new RRT during 30 days, and/or no renal recovery (defined as a ratio of Screat [the last recorded Screat before day 30 or ICU discharge if occurred before] to baseline Screat > 150%) [14].

We used Screat at admission (Screat_adm_) as baseline Screat except if eGFR at admission was < 75 ml/min and minimal creatinine dosage during ICU hospitalization (without RRT or history of CKD) was inferior to Screat_adm_. In that case we back calculated baseline Screat from the CKD-EPI equation set to 75 ml/min per 1.73 m^2^.

Blood was sampled within 24 h after admission. Samples were subsequently processed and stored at − 80 °C before transfer to the central laboratory for the blinded Gal-3 and plasmatic Neutrophil Gelatinase Associated Lipocalin (pNGAL) dosages.

Gal-3 and pNGAL were measured using a commercially available chemiluminescence immunoassay with ARCHITECT i2000 (Abbott Diagnostics, Abbott Park, IL) [15, 16]. Both, intra-assay and inter-assay coefficients of variation, as well as inter-individual variability (biological variation) for Gal-3 and pNGAL, with a commercially available chemiluminescence immunoassay (ARCHITECT i2000, Abbott Diagnostics, Abbott Park, IL) are reported to be low (i.e., < 6% for Gal-3 and < 3% for pNGAL) [15].

25th percentile, 50th percentile, 75th percentile, 95th percentile and 97th percentile where respectively in healthy population reported for Gal-3  as: 9.2 (8.8–9.6) ng/ml, 13.1 (12.4–13.8) ng/ml, 15.3 (14.5–16.5) ng/ml, 17.2 (15.8–17.8) ng/ml, and 17.9 (17.3–21.0) ng/ml [16]. For pNGAL, level quartile in healthy population were reported as: < 158 μg/l for Q1, 158–202 μg/l for Q2, 202–265 μg/l for Q3 and > 265 μg/l for Q4 [17]

### Endpoint

The primary endpoint was the renal prognostic evaluation assessed with MAKE at 30 days after ICU admission. The secondary endpoints were mortality at 30 days after ICU admission, AKI during 7 days after admission, no renal recovery.

### Statistical analysis

This report follows the Strengthening the Reporting of Observational studies in Epidemiology statement (STROBE) [18]. Continuous data were described as mean (standard deviation) for normal distributed variables and compared for bivariate analysis using a student T-test or as median [first (Q1), third (Q3) quantile] for the other variables and compared for bivariate analysis using Wilcoxon test. Categorical variables were expressed as count (percentages) and bivariate comparison was assessed using a Chi2 test or Kruskal–Wallis test for multivariate analysis (adjusted with Dunn’s method). A pairwise comparison was performed using Pearson's Chi-squared test and adjusted with Bonferroni method. Correlation ratio R-square was calculated using the Spearman test to assess monotonic relation between continuous variable and display in a logarithm or continuous scale. In order to determine relationship between levels of Gal-3 and outcome, we performed restricted cubic splines to explore the linearity of the association between biomarkers and outcome, and receiver operating characteristics curves for predictive performance. Optimal threshold was assessed using the best area under the received operating characteristic curve (AUROC) and described with optimal threshold value, sensitivity, specificity and accuracy. Comparison of prediction performance was assessed using reclassification method, expressed with continuous net reclassification improvement (NRI) and indexed discrimination improvement (IDI). Comparison of AUROC was performed using the Delong test. The Confidence interval was calculated using bootstrap. The Association between Gal-3 and outcome was assessed using binomial logistic regression, estimation values were expressed using odds ratio (OR CI_95%_). Multivariable analysis previously identified independent predictors of MAKE, which served as prognostic factors used for adjustment of logistic models: gender, age, vasopressor treatment, SAPS II, chronic renal disease, Charlson score, Screat at admission, lactate value at admission; models are assessed with or without pNGAL as adjustment variable.

Sensitivity analyses were also performed in subgroups of AKI, non-AKI patients and after exclusion of patients with chronic comorbidities (i.e., chronic heart failure [CHF], chronic kidney disease [CKD]). All reported probability values are two tailed, and *P* < 0.05 was considered statistically significant. All missing data were included in the analysis. Analyses were performed using R software (version 4.0; http://www.R-project.org) software and sensitivity analysis was performed using PredictABEL® package.

## Results

### Characteristics of patients

Two thousand and seventy-six patients included had Gal-3 levels at admission. Characteristics of patients are detailed in Table 1. The median age was 63 [51, 74] years and 723 (34.8%) were female. Median SAPS II score at admission was 49 [36, 63], 1600 (77.1%) patients received vasopressors. Median ICU length of stay was 13 [7, 21] days and median hospitalization length of stay was 23 [13, 39] days (Table 1). Three hundred and twenty-seven (15.9%) had admission diagnosis of shock (sepsis excluded) and/or cardiac arrest, 144 (7%) had acute heart failure (AHF) and 532 (25.9%) had severe sepsis or septic shock. One hundred fifty-two patients (7.3%) had a history of CHF, 240 (11.6%) of CKD, and 898 (43.3%) had chronic hypertension (Table 1). Median Screat_adm_ was 84 [59, 150] µmol/l, median Gal-3 dosage was 21 [14.2, 33] ng/ml, median plasma NGAL (pNGAL) was 209 [97, 506.8] µg/l and 774 (37.3%) patients had AKI. Characteristics of patients according to Gal-3 median dosage are shown in Additional file 1: Table S1.

**Table 1:** Characteristic table of patients, comparison between MAKE and NO MAKE and comparison between survivor and non-Survivor

	Overall (*N* = 2076)	NO MAKE (*N* = 1269)	MAKE (*N* = 807)	*P* value	Survivor (*N* = 1611)	Non-survivor (*N* = 465)	*P* value
Age—years (median [Q1, Q2])	63 [51, 74]	60 [48, 71]	68 [56.0, 77]	< 0.001	61 [48, 72]	70 [60, 79]	< 0.001
Female (%)	723 (34.8)	473 (37.3)	250 (31)	0.004	575 (35.7)	148 (31.8)	0.137
BMI (median [Q1, Q2])	26.5 [23.1, 30.8]	26 [22.8, 30.1]	27.3 [24.1, 31.2]	< 0.001	26.3 [23, 30.6]	27.1 [23.9, 31.1]	0.117
SOFA admission – score (median [Q1, Q2])	8 [5, 10]	7 [4, 10]	9 [6, 11]	< 0.001	7 [4, 10]	9 [6, 11]	< 0.001
SAPS II admission – score (median [Q1, Q2])	49 [36, 63]	44 [33, 58]	56 [43, 70]	< 0.001	46 [34, 59]	60 [46, 73]	< 0.001
Mechanical ventilation at admission (%)	1938 (93.4)	1190 (93.8)	748 (92.7)	0.380	1511 (93.8)	427 (91.8)	0.164
*Admission diagnostic *				< 0.001			< 0.001
Other (%)	456 (22.2)	351 (27.8)	105 (13.2)		406 (25.3)	50 (11.0)	
Shock and cardiac arrest (%)	327 (15.9)	162 (12.8)	165 (20.8)		225 (14)	102 (22.4)	
Acute cardiac failure (%)	144 (7)	81 (6.4)	63 (7.9)		108 (6.7)	36 (7.9)	
Acute respiratory failure (%)	392 (19)	262 (20.7)	130 (16.4)		305 (19)	87 (19.1)	
Secondary to surgery (%)	207 (10.1)	139 (11)	68 (8.6)		171 (10.7)	36 (7.9)	
Severe sepsis (%)	532 (25.9)	269 (21.3)	263 (33.1)		388 (24.2)	144 (31.6)	
*Comorbidities*							
Chronic heart failure (%)	152 (7.3)	68 (5.4)	84 (10.4)	< 0.001	102 (6.3)	50 (10.8)	0.002
Diabetes mellitus (%)	383 (18.5)	196 (15.5)	187 (23.2)	< 0.001	281 (17.5)	102 (22.0)	0.033
Chronic hypertension (%)	898 (43.3)	465 (36.7)	433 (53.7)	< 0.001	640 (39.8)	258 (55.6)	< 0.001
Chronic dyslipidemia (%)	409 (19.7)	230 (18.2)	179 (22.2)	0.028	315 (19.6)	94 (20.3)	0.800
Chronic peripheral vascular disease (%)	207 (10)	102 (8.1)	105 (13)	< 0.001	144 (9)	63 (13.6)	0.005
Chronic COPD (%)	272 (13.1)	149 (11.8)	123 (15.3)	0.026	192 (11.9)	80 (17.2)	0.004
Chronic liver disease (%)	158 (7.6)	82 (6.5)	76 (9.4)	0.017	96 (6)	62 (13.4)	< 0.001
Chronic renal disease (%)	240 (11.6)	87 (6.9)	153 (19)	< 0.001	158 (9.8)	82 (17.7)	< 0.001
Chronic malignant tumor (%)	279 (13.5)	143 (11.3)	136 (16.9)	< 0.001	191 (11.9)	88 (19)	< 0.001
Chronic inflammatory disease (%)	77 (3.7)	51 (4)	26 (3.2)	0.411	63 (3.9)	14 (3)	0.445
*Chronic treatment*							
Aldosterone agonist (%)	14 (0.7)	6 (0.5)	8 (1)	0.258	6 (0.4)	8 (1.7)	0.005
Diuretics (%)	447 (21.7)	219 (17.4)	228 (28.5)	< 0.001	304 (19.0)	143 (31)	< 0.001
ACE inhibitors or angiotensin II receptor blockers (%)	552 (26.8)	292 (23.2)	260 (32.5)	< 0.001	402 (25.2)	150 (32.5)	0.002
*Physiological admission parameters*							
Systolic blood pressure – mmHg (median [Q1, Q2])	122 [108, 139]	124 [110, 140]	120 [105, 136]	< 0.001	123 [109, 140]	118 [104.5, 133]	< 0.001
Diastolic blood pressure—mmHg (median [Q1, Q2])	61 [53, 70]	63 [55, 72.2]	58 [51, 67]	< 0.001	62 [55, 72]	56 [50, 65]	< 0.001
Diuresis during the first 24 h – ml (median [Q1, Q2])	1350 [800, 2200]	1545 [1000, 2400]	903.5 [355, 1700]	< 0.001	1425 [900, 2300]	903.5 [400, 1688]	< 0.001
*Biological admission parameters*							
Admission plasmatic creatinine—µmol/l (median [Q1, Q2])	84 [59, 150]	70.2 [54, 102.7]	142.8 [80, 226]	< 0.001	78 [57, 134]	116 [74, 193]	< 0.001
Admission plasma lactate dosage—mmol/l (median [Q1, Q2])	1.4 [1, 2]	1.4 [1, 1.8]	1.6 [1.1, 2.4]	< 0.001	1.4 [1, 1.9]	1.6 [1.2, 2.5]	< 0.001
Galectin-3 admission dosage – ng/ml (median [Q1, Q2])	21 [14.2, 33]	16.9 [12.7, 24.3]	30.2 [20.8, 49.2]	< 0.001	18.8 [13.3, 29.2]	29.2 [20.2, 49.2]	< 0.001
NGAL admission dosage – µg/l (median [Q1, Q2])	209 [97, 506.8]	139 [80, 275]	483 [209.8, 904.8]	< 0.001	173 [86, 418.2]	410 [181.8, 866.2]	< 0.001
*Renal outcomes and function*							
MAKE (%)	807 (38.9)	-	-		342 (21.2)	465 (100)	< 0.001
No AKI (%)	1302 (62.7)	1048 (82.6)	254 (31.5)	< 0.001	1079 (67)	223 (48)	< 0.001
AKI (%)	774 (37.3)	221 (17.4)	553 (68.5)	< 0.001	532 (33)	242 (52)	< 0.001
KDIGO 1 (%)	245 (11.8)	153 (12.1)	92 (11.4)		189 (11.7)	56 (12)	
KDIGO 2 (%)	119 (5.7)	45 (3.5)	74 (9.2)		82 (5.1)	37 (8)	
KDIGO 3 (%)	410 (19.7)	23 (1.8)	387 (48)		261 (16.2)	149 (32)	
*General outcomes*							
Mortality (%)	465 (22.4)	0 (0)	465 (57.6)	< 0.001	-	-	
In ICU length of stay – days (median [Q1, Q2])	13 [7, 21]	12 [7, 20]	14 [8, 24]	< 0.001	13 [7, 24]	11 [7, 17]	< 0.001
Hospital Length of stay – days (median [Q1, Q2])	23 [13, 39]	26 [15, 42]	18 [10, 31]	< 0.001	28.0 [16, 46]	12.0 [7, 19]	< 0.001
*Treatments during hospitalization*							
Vasopressors (%)	1600 (77.1)	896 (70.6)	704 (87.2)	< 0.001	1188 (73.7)	412 (88.6)	< 0.001
Renal replacement therapy (%)	374 (18)	0 (0)	374 (46.3)	< 0.001	237 (14.7)	137 (29.5)	< 0.001

BMI, body mass index; SOFA, Sequential organ failure assessment; SAPSII, Simplified acute physiology score 2; COPD, Chronic obstructive pulmonary disease; ACE, Angiotensin-converting enzyme; AKI, Acute kidney injury; KDIGO, Kidney Disease, Improving Global Outcomes; ICU, intensive care unit; MAKE, Major adverse kidney event

### Gal-3 at admission is associated with MAKE

**Fig. 1:**
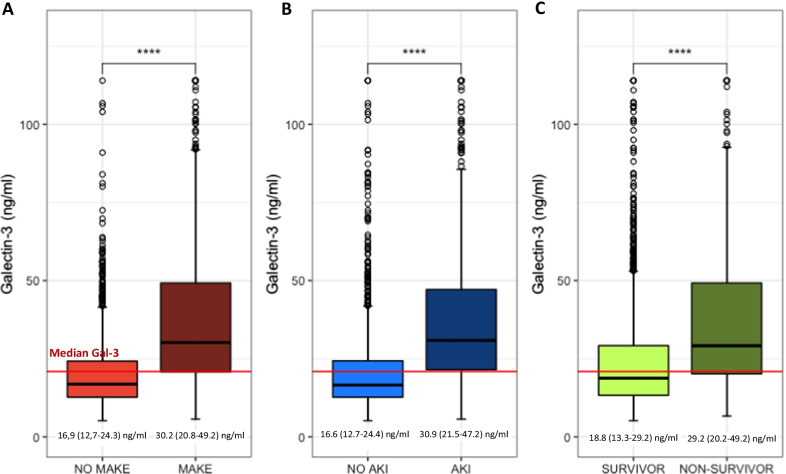
Gal-3 level for MAKE and NO MAKE (**A**), Gal-3 level for AKI and NO AKI patients (**B**), Gal-3 level for survivor and non-survivor patients (**C**). MAKE: Major adverse kidney event, AKI: Acute kidney injury. *p* value: ns > 0.05, *0.05–0.01; **0.01–0.001; ***< 0.001

Eight hundred and seven (38.9%) patients developed MAKE. Patients who developed MAKE had higher Gal-3 levels on admission (30.2 [20.8, 49.2] ng/ml versus 16.9 [12.7, 24.3] ng/ml, *p* < 0.001, respectively) (Fig. 1A and Table 1).

**Fig. 2:**
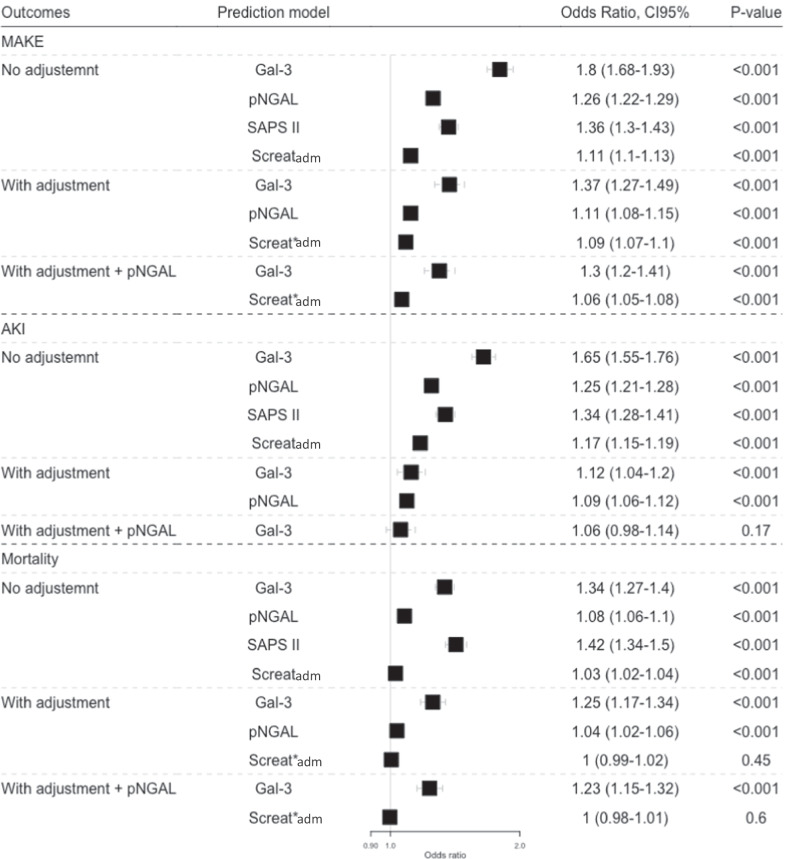
Gal-3, SAPSII, Screat_adm_, pNGAL, Gal-3 after adjustment and Screat_adm_ after adjustment, Gal-3 after adjustment including pNGAL and Screat_adm_ after adjustment including pNGAL association with MAKE, AKI and Mortality. Gal-3 association was adjusted with gender, age, CKD, vasopressor treatment, SAPS II, Charlson score, Screat_adm_ and lactate value at admission. For AKI association, Screat was not include in the adjustment model. OR for continuous variables (Gal-3, SAPSII, Screat_adm_) were standardized for each 10-unit change, and pNGAL for each 100-fold unit change. *These model do not include Screat_adm_. OR, odds ratio; CI, confidence interval; SAPS II, Simplified acute physiology score II; MAKE, Major adverse kidney event; Gal-3, galectin 3; Screat, serum creatinine; CKD, Chronic kidney disease

Plasma Gal-3 was associated with the risk of MAKE (OR 1.80 CI_95%_ [1.68–1.93], *p* < 0.001, non-adjusted and OR 1.37 CI_95%_ [1.27–1.49], *p* < 0.001, adjusted) (Fig. 2). The different contributors of MAKE are presented in Additional file 1: Fig. S1. Gal-3 was associated with all components of MAKE, including mortality (OR 11.54 CI_95%_ [6.20–21.48]) and non-renal recovery only (OR 6.13 CI_95%_ [4.12–9.10]) (Additional file 1: Fig. S2A).

**Fig. 3:**
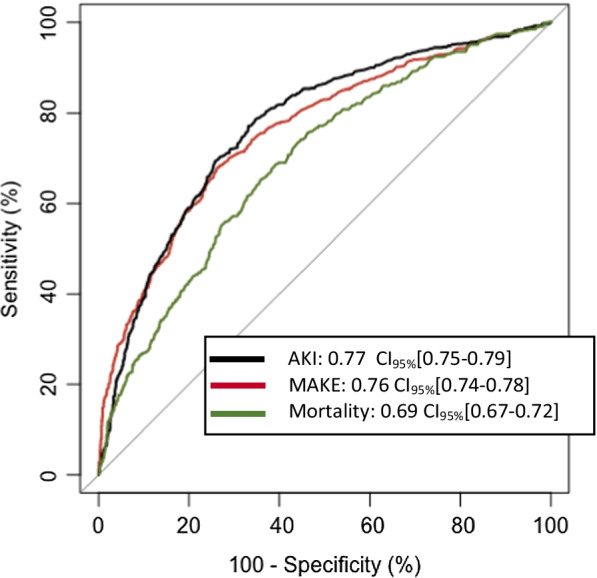
ROC curve of Gal-3 prediction performance for MAKE, AKI and mortality. AKI, Acute kidney injury; ROC, receiver operating characteristics; CI, confidence interval; MAKE, Major adverse kidney event

The AUROC of Gal-3 for MAKE prediction was 0.76 CI_95%_ [0.74–0.78], *p* < 0.001 (Fig. 3), sensitivity, specificity and accuracy are described in Additional file 1: Table S2, and the prediction performance was significantly higher than Screat_adm_ (vs AUROC of 0.74 CI_95%_ [0.71–0.76], *p* < 0.001) for MAKE prediction (*p* = 0.04 and added to the performance of Screat_adm_ or Screat_adm_ + SAPS II (Additional file 1: Fig. S3A). Gal-3 and pNGAL had no different prediction performance for MAKE (vs AUROC of 0.77 [0.75–0.79], *p* = 0.42, but Gal-3 performed better than CRP or lactate (vs AUROC of 0.60 [0.58–0.63], *p* < 0.001 and vs AUROC of 0.55 [0.53–0.58], *p* < 0.001, respectively) (Additional file 1: Fig. S4A). The use of Gal-3 added prediction performance. Performance of pNGAL for primary endpoint and implication of model improvement represented with continuous Net Reclassification Improvement (NRI) and Integrated Dyiscrimination improvement are presented in Additional file 1: Table S3 and Fig. S5.

**Fig. 4:**
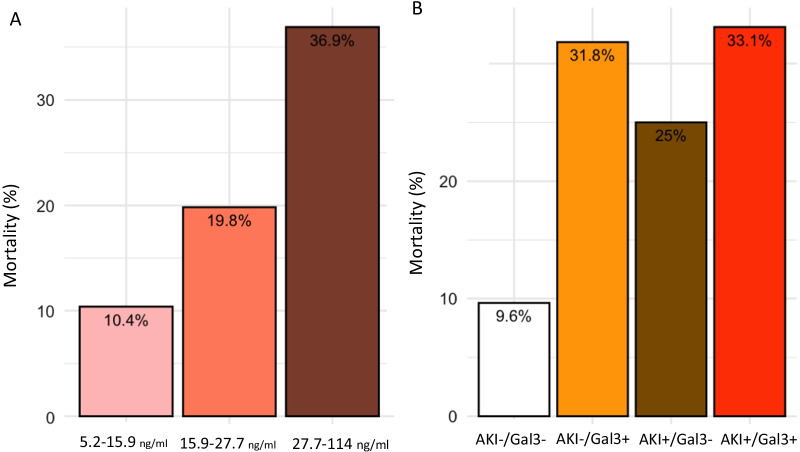
Prevalence of mortality according to level of Gal3 (tercile stratification) (**a**). Prevalence of mortality according to Gal3 positive biomarker and AKI positive biomarker (**b**). Gal-3, galectin 3; AKI, acute kidney injury

The association and prediction performance for MAKE remained among patients with AKI (OR CI_95%_ 1.39 CI 95% [1.26–1.54], *p* < 0.001, non-adjusted and OR 1.31 CI_95%_ [1.19–1.46], *p* < 0.001, adjusted (Additional file 1: Fig. S6A, S7A, S9A). Furthermore, Gal-3 improved the risk stratification when combined with Screat_adm_ (i.e., AKI, Fig. 4). Among patient with AKI, Gal-3 level was significantly higher among patients with no renal recovery than among patients with renal recovery (35 [24.8, 55.4] ng/ml versus 27.5 [20.1, 40.3] ng/ml, *p* < 0.001, respectively) (Additional file 1: Fig. S10 and Table S4). Gal-3 remained associated with non-renal recovery after adjustment for potential confounding factors (adjusted with gender, age, CKD, vasopressor treatment, SAPS II, Charlson score, Screat and lactate at admission), OR 1.13 [1.05–1.22] (Additional file 1: Fig. S2C). Gal-3 remained associated with MAKE after adjustment including pNGAL with an OR 1.30 CI_95%_ [1.20, 1.41], *p* > 0.001 (Fig. 2).

### Increased Gal-3 at admission in patients with AKI

**Fig. 5:**
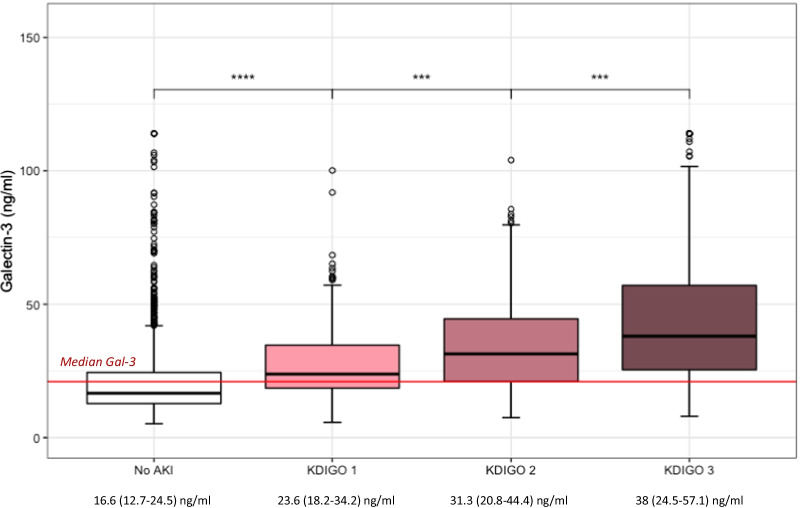
Gal-3 level according to each acute kidney injury (AKI) stage of the KDIGO classification. AKI, acute kidney injury; KDIGO, Kidney Disease Improving Global Outcomes; ns, non-significant. *P* value: ns: > 0.05, *0.05–0.01; **0.01–0.001; ***> 0.001

One thousand three hundred and two (62.7%) patients had no AKI with a median Gal-3 of 16.6 [12.7, 24.5] ng/ml, 245 (11.8%) had stage 1 AKI with a median Gal-3 of 23.6 [18.2, 34.2] ng/ml, 119 (5.7%) had stage 2 AKI with Gal-3 of 31.3 [20.8, 44.4] ng/ml, 410 (19.7%) had stage 3 AKI with Gal-3 of 38 [24.5, 57.1] ng/ml (Table 1 and Fig. 5). Gal-3 was associated with AKI; OR 1.65 CI_95%_ [1.55–1.76], *p* < 0.001, non-adjusted, OR 1.12 CI_95%_ [1.04–1.20], *p* < 0.001, adjusted (Fig. 2).

Gal-3 was inversely correlated with eGFR (rho = − 0.69, *p* < 0.001) and median Gal-3 significantly increased with stratified eGFR (*p* < 0.001) (Additional file 1: Fig. S11).

Predictive performance of Gal-3 for AKI with an AUROC of 0.77 CI_95%_ [0.75–0.79] and was significantly higher than SAPS II, CRP or lactate. pNGAL had significantly AUROC for AKI (vs 0.81 [0.79–0.83], *p* > 0.001) (Fig. 3, Additional file 1: Fig. S4 and S5).

### Gal-3 at admission is associated with mortality at 30 days

Four hundred and fifty-five (22.4%) patients died within 30 days. Median Gal-3 was higher in non-survivors compared to survivors (29.2 [20.2, 49.2] ng/ml versus 18.8 [13.3, 29.2] ng/ml, *p* < 0.001, respectively) (Table 1 and Fig. 1C). The risk of MAKE and death appeared to increase with Gal-3 level above 20 ng/ml (Additional file 1: Fig. S12).

Gal-3 was associated with mortality with an OR 1.34 CI_95%_ [1.37–1.40], *p* < 0.001, non-adjusted and OR 1.25 CI_95%_ [1.17–1.34], *p* < 0.001, adjusted (Fig. 2). The AUROC of Gal-3 for 30 days mortality prediction was higher than Screat_adm_ or SAPS II (0.69 CI_95%_ [0.67–0.72] for Gal-3 vs. 0.63 CI_95%_ [0.60–0.66] for Screat_adm_ and 0.69 CI_95%_ [0.66–0.71] for SAPS II, *p* < 0.001) (Fig. 3 and Additional file 1: Fig. S3). The association remained in the sensitivity analysis among non-AKI patients (OR 1.64 CI_95%_ [1.49–1.82], *p* < 0.001, non-adjusted, OR 1.44 (1.30–1.62), *p* > 0.001, adjusted) (Additional file 1: Fig. S6B, S8 and S9C), and among AKI patient after adjustment (OR 1.13 CI_95%_ [1.06–2.21], *p* < 0.001, non-adjusted, OR 1.09 CI_95%_ [1.01–1.17], *p* < 0.001, adjusted, Additional file 1: Fig. S6A, S7B, S9A) and after excluding patients with CHF or CKD (Additional file 1: Fig. S13).

## Discussion

In this cohort study, Gal-3 was associated with MAKE and all its components (i.e., mortality at day 30, RRT and non-recovery at day 30 after admission to the ICU). Gal-3 levels increased with AKI severity and correlated with minimal eGFR during the first 7 days after admission. Altogether, these data suggest that Gal-3 might be a mediator involved in poor outcomes, especially in patients with AKI, and improve the risk stratification compared to Screat_adm_ (i.e., meeting the AKI criteria). Gal-3 may serve as a biomarker for predictive and prognostic enrichment in critical care trials.

In the present study, we explored the prognostic value of Gal-3 at admission in a mixed ICU population. We found that Gal-3 was strongly associated with AKI severity and was associated with short-term outcomes, i.e., MAKE and mortality. In line with previous preclinical studies, the timeline of the association between elevated Gal-3 and outcomes (i.e., MAKE, death) is coherent with the induction of regional and systemic inflammatory response and induction of profibrotic pathways by the biomarker. Interestingly, Gal-3 had better predictive performance of MAKE in non-AKI population compared to AKI patients. Gal-3 however improved the risk stratification of patients compared to Screat_adm_ (i.e., AKI criteria). The relationship between Gal-3 and AKI is complex. Gal-3 appears to both contribute to AKI and increase in response to AKI. Gal-3 has been shown to be associated with kidney injury [7]. There is an obvious collinearity between increased Screat_adm_ (and the diagnosis of AKI). This may contribute to lower performance of Gal-3 in AKI patients (*in other words,* AKI itself being a strong predictor of poor outcomes). Preoperative serum Gal-3 was shown to be associated with postoperative AKI or mortality after cardiac surgery [19, 20]. However, these studies did not explore renal outcomes.

Gal-3 is 30kDA size protein and is expected to be partly cleared by the kidney [3]. In rats, Gal‐3 plasma clearance was 0.92 mL/min. In humans, renal Gal‐3 clearance was reported to be 3.9 mL/min [2.3–6.4] mL/min in healthy subjects and 2.3 mL/min [1.5–3.4] ml/min in heart failure (HF) patients. Meijers et al. observed that creatinine clearance was inversely correlated with plasma Gal‐3 levels (rho = − 0.315, *P* = 0.001) [21]. Of note in our study, Gal-3 level was measured at admission, before renal replacement therapy initiation therefore RRT did not impact Gal-3 levels.

Gal-3 is an ubiquitous lectin expressed in multiple organs [3]. Increased Gal-3 levels have been associated with adverse clinical outcomes in the general population and in patients with chronic cardiovascular diseases [22]. The important role of Gal‐3 in HF was first described by Sharma et al. [5], which reported that this lectin was the strongest differentially regulated gene associated with HF. Subsequently, an increased level of myocardial Gal‐3 has been observed in several animal models of heart disease [5, 6, 22, 23] and in clinical settings [24–27]. Gal-3 is mainly expressed in activated macrophages and pathologically damaged cardiomyocytes and is considered as an active contributor to cardiac remodeling, including myocardial fibrogenesis, and to the development of HF [5]. Gal-3 was also shown to play a role in nephrogenesis as it is upregulated with fetal kidney maturation [28, 29] and in diabetic nephropathy, as it functions acts an advanced glycation end products (AGE) receptor in vivo thereby providing protection against AGE-dependent tissue injury [30]. An association between higher levels of plasma Gal-3 and a rapid decline in eGFR was observed in patients with CKD [31]. Circulating Gal-3 levels increased in parallel with decreasing kidney function and were markedly elevated in patients with end stage diabetes [32] and were significantly associated with cardiovascular events or mortality [33]. Identification of the role of Gal-3 in acute settings is more recent [8]. While a contribution of chronic comorbidities in the Gal-3 rises and association with outcome cannot be excluded in our study, the persistent association despite exclusion of patients with CHF or CKD strongly suggests a role of acute illness in Gal-3 expression. Moreover, Gal-3 remained associated with non-renal recovery, even after adjustment using known associated factors (i.e., AKI severity, CKD, sepsis, vasopressor treatment, emergency admission) (Additional file 1: Fig. S2C).

Recent studies explored the impact of Gal-3 inhibitors to prevent kidney injury or improve post-AKI outcomes [8, 25, 34, 35]. Prud’homme et al. showed that experimental AKI increased both renal and cardiac expression of Gal-3 originating from bone-marrow-derived cells and promoted cardiac injury after AKI [8]. Genetic or pharmacological inhibition of Gal-3 prevented AKI-induced cardiac injury, inflammation, fibrosis, and cardiac dysfunction [8]. More recently, Gal-3 expression was highly increased in septic AKI and the use of Gal-3 inhibitors in a septic model reduced mortality in rats [9].

We acknowledge the limitations of this study. First, baseline Screat was not available and admission Screat was used to define baseline Screat for most patients. Secondly, single measurement of Gal-3 was available at inclusion without trends. Finally, as an observational cohort study showing the association between Gal-3 and prognosis, the causal relationship between activation of the Gal-3 pathway and prognosis cannot be confirmed. In our opinion, these data provide a strong argument for testing the impact of Gal-3 inhibition on outcomes in critically ill patients and elevated plasma Gal-3 levels.

Altogether, these data suggest that Gal-3 is strongly associated with AKI and could improve the risk stratification for the risk of death and MAKE. This biomarker improves classification of patient for poor outcomes. This adds to the existing literature Gal-3 as a key contributor of poor renal outcomes. Strategies inhibiting this lectin should be now tested in this setting. Finally, incorporating Gal-3 in predictive models would be able to improve accuracy of the model and better identify patients at risk of poor outcomes who may benefit from targeted interventions after ICU admission. The observed association with outcomes after adjustment for other strong predictive factors such as lactate and pNGAL suggests that Gal-3 provides additional information. Therefore, inhibition of the Gal-3 pathway appears a promising strategy to improve outcomes in critically ill patients with kidney damages.

## Conclusion

Gal-3 at ICU admission is associated with poor renal and global outcomes (i.e., MAKE) and mortality at day 30 after admission. Gal-3 is also strongly associated with AKI severity. The results of this study should encourage the use of Gal-3 as a biomarker for predictive and prognostic enrichment in AKI-related trials and test the impact of Gal-3 inhibition on outcomes in critically ill patients and elevated plasma Gal-3 levels.

## Supplementary Information

**Additional file 1**:** Fig. S1–S13 and Table S1–S4**. Elevated plasma Galectin-3 is associated with major adverse kidney events and death after ICU admission - Additional data.

## Data Availability

All authors had full access to all data in the study and takes responsibility for the integrity of the data.

## Abbreviations

AKI: Acute kidney injury
AUROC: Area under the receive operating characteristic
AGE: Antigen glycation ends
ICU: Intensive care unit
MAKE: Major adverse kidney event
Gal-3: Galectin-3
KDIGO: Kidney disease improvement of globe outcome
ADQI: Acute Disease Quality Initiative
SOFA: Sequential Organ Failure Assessment
SAPS II: Simplified Acute Physiology Score II
Screat: Serum creatinine
