# Supplementary material for: Elevated plasma Galectin-3 is associated with major adverse kidney events and death after ICU admission
Source: Crit Care. 2022 Jan 6;26:13. doi: 10.1186/s13054-021-03878-x (PMC8740042; doi:10.1186/s13054-021-03878-x)
Supplement: Supplementary file 1 — Additional file 1: Fig. S1–S13 and Table S1–S4. Elevated plasma Galectin-3 is associated with major adverse kidney events and death after ICU admission - Additional data. [file 13054_2021_3878_MOESM1_ESM.docx]

|  | Under median Gal-3  (N=1038) | | Over median Gal-3  (N=1038) | P-value |
| --- | --- | --- | --- | --- |
| Age - years (median [Q1, Q2])) | 58 [45.2, 70] | 67 [56.2, 77] | | <0.001 |
| Female (%) | 361 (34.8) | 362 (34.9) | | 1.000 |
| BMI (median [Q1, Q2]) | 25.8 [22.7, 29.3] | 27.3 [23.9, 31.9] | | <0.001 |
| SOFA admission – score (median [Q1, Q2]) | 6 [4, 9] | 9 [6.0, 11] | | <0.001 |
| SAPS II admission – score (median [Q1, Q2]) | 42 [32, 56] | 55 [41.2, 69] | | <0.001 |
| Mechanical ventilation at admission (%) | 983 (94.7) | 955 (92) | | 0.017 |
| **Admission diagnostic (%)** |  |  | | <0.001 |
| Other (%) | 323 (31.1) | 133 (13) | |  |
| Shock and cardiac arrest (%) | 163 (15.7) | 164 (16.1) | |  |
| Acute cardiac failure (%) | 55 (5.3) | 89 (8.7) | |  |
| Acute respiratory failure (%) | 194 (18.7) | 198 (19.4) | |  |
| Secondary to surgery (%) | 108 (10.4) | 99 (9.7) | |  |
| Severe sepsis (%) | 194 (18.7) | 338 (33.1) | |  |
| **Comorbidities** |  |  | |  |
| Chronic heart failure (%) | 45 (4.3) | 107 (10.3) | | <0.001 |
| Diabetes mellitus (%) | 136 (13.1) | 247 (23.8) | | <0.001 |
| Chronic hypertension (%) | 340 (32.8) | 558 (53.9) | | <0.001 |
| Chronic dyslipidemia (%) | 171 (16.5) | 238 (23) | | <0.001 |
| Chronic peripheral vascular disease (%) | 79 (7.6) | 128 (12.4) | | <0.001 |
| Chronic COPD (%) | 117 (11.3) | 155 (15) | | 0.016 |
| Chronic liver disease (%) | 54 (5.2) | 104 (10) | | <0.001 |
| Chronic renal disease (%) | 32 (3.1) | 208 (20.1) | | <0.001 |
| Chronic malignant tumor (%) | 112 (10.8) | 167 (16.1) | | 0.001 |
| Chronic inflammatory disease (%) | 30 (2.9) | 47 (4.5) | | 0.063 |
| **Chronic treatment** |  |  | |  |
| Aldosterone agonist (%) | 4 (0.4) | 10 (1) | | 0.177 |
| Diuretics (%) | 151 (14.6) | 296 (28.8) | | <0.001 |
| ACE inhibitors or angiotensin II receptor blockers (%) | 229 (22.2) | 323 (31.5) | | <0.001 |
| **Physiological admission parameters** |  |  | |  |
| Systolic blood pressure – mmHg (median [Q1, Q2]) | 125 [110, 141] | 120 [106, 137] | | <0.001 |
| Diastolic blood pressure – mmHg (median [Q1, Q2]) | 64 [56, 73] | 58 [51, 67] | | <0.001 |
| Diuresis during the first 24 hours – ml (median [Q1, Q2]) | 1500 [1000, 2300] | 1130 [500, 2000] | | <0.001 |
| **Biological admission parameters** |  |  | |  |
| Admission plasmatic creatinine - µmol/l (median [Q1, Q2]) | 65 [51, 82] | 142 [87, 215] | | <0.001 |
| Admission plasma lactate dosage - mmol/l (median [Q1, Q2]) | 1.3 [1, 1.8] | 1.5 [1.1, 2.3] | | <0.001 |
| Galectin-3 admission dosage – ng/ml (median [Q1, Q2]) | 14.2 [11.4, 17] | 33 [25.5, 47.7] | | <0.001 |
| NGAL at admission dosage – µg/l (median [Q1, Q2]) | 109 [67, 204] | 419 [210, 822] | | <0.001 |
| **Renal Outcomes** |  |  | |  |
| MAKE (%) | 207 (19.9) | 600 (57.8) | | <0.001 |
| Renal function |  |  | |  |
| No AKI (%) | 862 (83) | 440 (42,4) | | <0.001 |
| AKI (%) | 176 (17) | 598 (57.6) | | <0.001 |
| KDIGO 1 (%) | 84 (8.1) | 161 (15.5) | |  |
| KDIGO 2 (%) | 30 (2.9) | 89 (8.6) | |  |
| KDIGO 3 (%) | 62 (6.0) | 348 (33.5) | |  |
| **General outcomes** |  |  | |  |
| Mortality (%) | 127 (12.2) | 338 (32.6) | | <0.001 |
| In ICU length of stay – days (median [Q1, Q2]) | 12 [7, 20] | 13 [7.2, 23] | | 0.005 |
| Hospitalization length of stay – days (median [Q1, Q2]) | 22 [13, 38] | 24 [13, 40.8] | | 0.370 |
| **Treatments during hospitalisation** |  |  | |  |
| Vasopressors (%) | 705 (67.9) | 895 (86.2) | | <0.001 |
| Renal replacement therapy (%) | 67 (6.5) | 307 (29.6) | | <0.001 |

**Supplementary Table 1**: Characteristics table of patient according to median Gal-3 level.

*BMI: body mass index, SOFA: Sequential organ failure assessment, SAPSII: Simplified acute physiology score 2, COPD: Chronic obstructive pulmonary disease, ACE: Angiotensin-converting enzyme, AKI: Acute kidney injury, KDIGO: Kidney Disease: Improving Global Outcomes, ICU: intensive care unit.*

**No renal recovery**


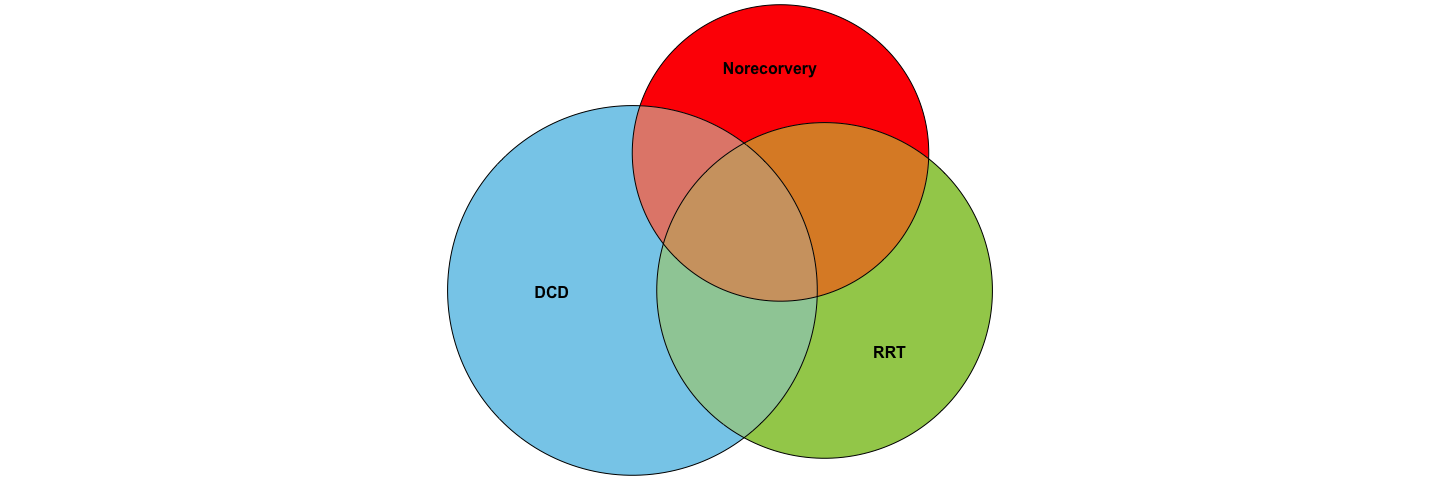


**N= 105 (13%)**

**N= 57 (7%)**

**No renal recovery + Mortality**

**RRT + No renal recovery**

**N= 89 (11%)**

**RRT + No renal recovery + Mortality**

**N= 52 (6.4%)**

**RRT**

**N= 148 (18.4%)**

**RRT + Mortality**

**N= 85 (10.6%)**

**Mortality**

**N= 271 (33.6%)**

**B.**

**A.**


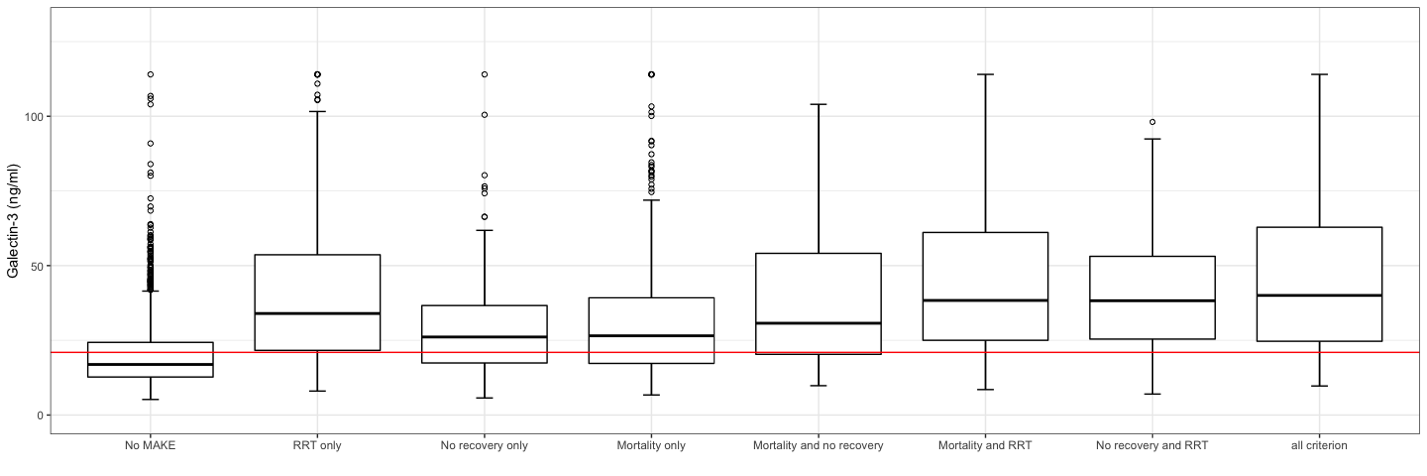


**Median Gal-3**

**No MAKE RRT only No renal recovery only Mortality only No Renal Recovery Mortality No Renal Recovery All criteria
 + Mortality + RRT + RRT**

**Supplementary Figure 1**: Number and percentage of MAKE subdivision **(A)** and Gal-3 level at admission among these MAKE subdivision **(B).**

*MAKE: Major adverse kidney event, RRT: Renal replacement therapy.*


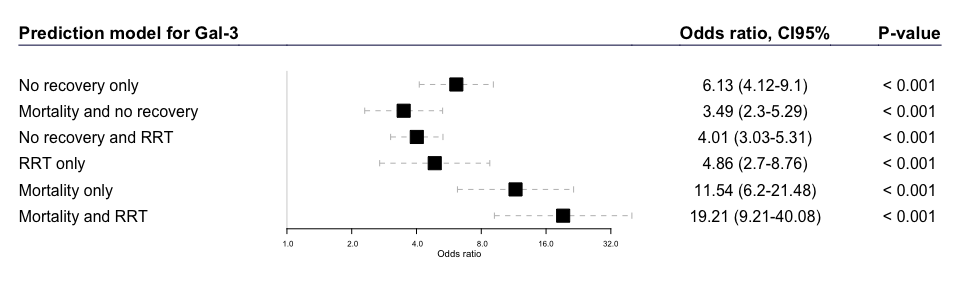


**A.**

**B.**


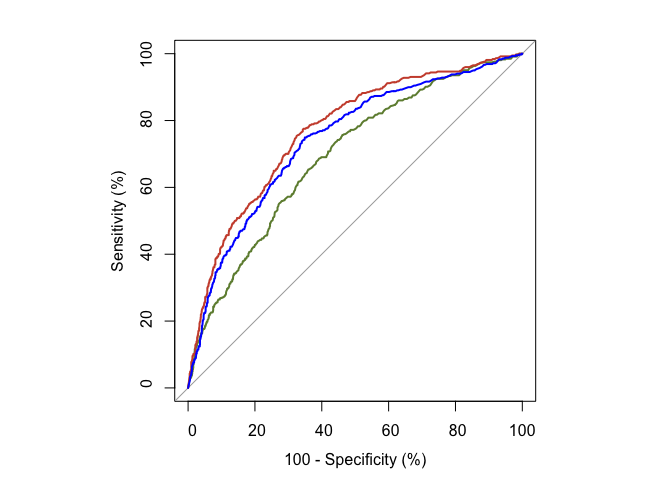


**C.**

Mortality: AUC 0,69 CI_95%_(0.67-0.72)

RRT: AUC 0,77 CI_95%_(0.74-0.8)

No recovery: AUC 0,74 CI_95%_(0.72-0.77)

**GAL3**

**
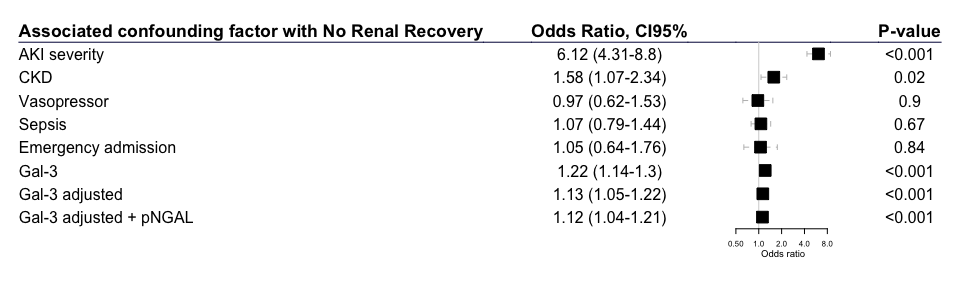
**

**Supplementary Figure 2:** Gal-3 association MAKE subdivision (**A)**, diagnostic performances for MAKE subdivision using Gal3 **(B),** confounding factors association with no renal recovery among AKI patients, Gal-3 association performance was adjusted using AKI severity, CKD, vasopressor therapy, sepsis, emergency admission. Gal-3 association was adjusted with gender, age, CKD, vasopressor treatment, SAPS II, Charlson score, Screat at admission and lactate value at admission **(C)**.

*MAKE: Major adverse kidney event, RRT: renal replacement therapy, CKD: Chronic kidney disease.*

|  | Threshold (ng/ml) | Specificity (%) | Sensitivity (%) | Accuracy (%) |
| --- | --- | --- | --- | --- |
| MAKE | | | | |
| Optimal value of Gal-3 | 23,8 | 73,6 | 68 | 71,4 |
| Median Gal-3 | 21 | 65,5 | 74,4 | 68,9 |
| AKI | | | | |
| Optimal value of Gal-3 | 21 | 66,8 | 77,1 | 70,7 |
| Median Gal-3 | 21 | 66,2 | 77,3 | 70,3 |
| Mortality | | | | |
| Optimal value of Gal-3 | 20 | 53,8 | 75,7 | 58,7 |
| Median Gal-3 | 21 | 56,6 | 72,7 | 60,2 |

**Supplementary Table 2:** Optimal values of Gal-3 prediction performance for MAKE, AKI and mortality.

*Gal-3: galectin 3, MAKE: Major adverse kidney event, AKI: Acute kidney injury*


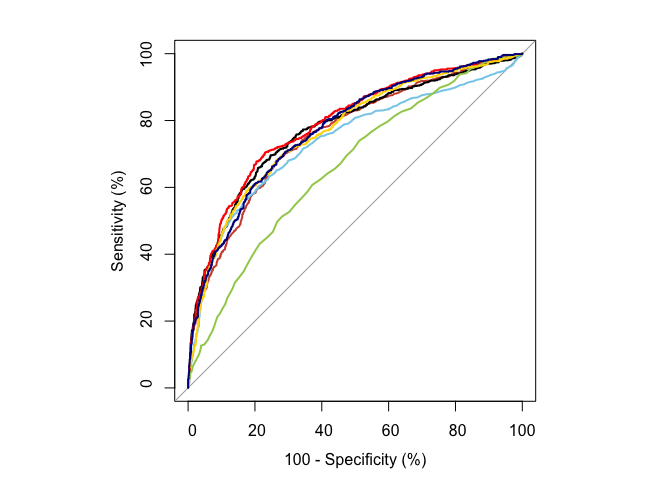


**A.**

**MAKE
All patients**

Gal-3: 0.76 CI_95%_[0.74-0.78]

SCreat_adm_: 0.74 CI_95%_[0.71-0.76]

SAPS II: 0.66 CI_95%_[0.64-0.68]

Gal-3+Screat_adm_: 0.78 CI_95%_[0.75-0.8]

Gal-3+Screat_adm_+SAPSII: 0.79 CI_95%_[0.77-0.81]

SAPS II+Screat_adm_: 0.77 CI_95%_[0.75-0.79]
 SAPS II+Gal-3: 0.77 CI_95%_[0.75-0.79]

*Gal-3 vs Screat_adm_, p-value =0.04*

*Gal-3 vs SAPSII, p-value < 0001*

*Gal-3 vs Gal-3+Screat_adm_, p-value <0.001*

*Gal-3 vs Gal-3 + Screat_adm_+SAPSII, p-value < 0.001*

*Gal-3 vs Screat_adm_+SAPSII, p-value = 0.39*

*Gal-3 vs SAPSII+Gal-3, p-value = 0.01*


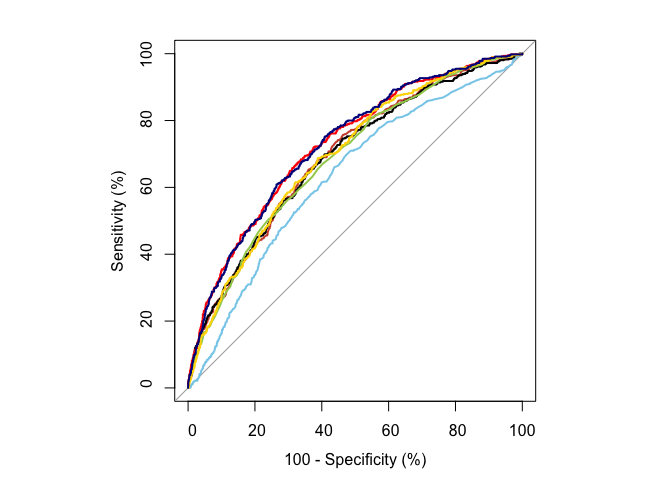


**B.**

**Mortality**

**All patients**

Gal-3: 0.69 CI_95%_[0.67-0.72]

SCreat_adm_: 0.63 CI_95%_[0.6-0.66]

SAPS II: 0.69 CI_95%_[0.66-0.71]

Gal-3+Screat_adm_: 0.69 CI_95%_[0.66-0.72]

Gal-3+Screat_adm_+SAPSII: 0.73 CI_95%_[0.7-0.75]

Screat_adm_+SAPSII: 0.69 CI_95%_[0.67-0.72]

SAPSII + Gal-3: 0.73 CI_95%_[0.70-0.75]

*Gal-3 vs Screat_adm_, p-value < 0.001*

*Gal-3 vs SAPS2, p-value = 0.7*

*Gal-3 vs Gal-3+Screat_adm_, p-value=0.13*

*Gal-3 vs Gal-3 + Screat_adm_,+SAPSII, p-value = 0.001*

*Gal-3 vs SAPSII+Screat_adm_, p-value=0.9*

*Gal-3 vs SAPSII+Gal-3, p-value = 0.001*

**Supplementary Figure 3:** ROC curve for MAKE **(A)** and mortality **(B)** performance prediction including using Gal-3, SAPSII and Screat_adm_ parameters.

*Gal-3: galectin 3, MAKE: Major adverse kidney event, SAPSII: Simplified acute physiology score, Screat_adm_: Serum creatinine at admission, ROC: Receiver operating characteristics*


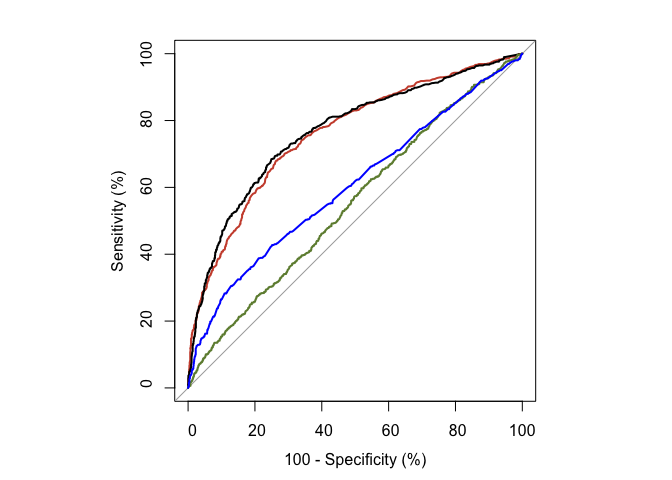


Comparison with Delong test

Gal-3 vs pNGAL: p-value = 0.42

Gal-3 vs Lactate, p-value <0.001

Gal-3 vs CRP, p-value < 0.001

MAKE

**B.**

**A.**

AUC Gal-3 : 0.76 (0.74-0.78)

AUC pNGAL : 0.77 (0.75-0.79)

AUC Lactate: 0.6 (0.58-0.63)

AUC CRP: 0.55 (0.53-0.58)


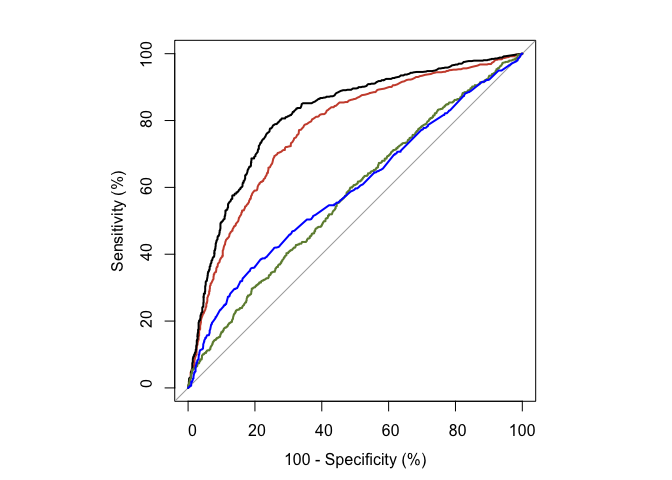


Comparison with Delong test

Gal-3 vs pNGAL: p-value <0.001

Gal-3 vs Lactate, p-value <0.001

Gal-3 vs CRP, p-value < 0.001

AKI

AUC Gal-3 : 0.77 (0.75-0.79)

AUC pNGAL : 0.81 (0.79-0.83)

AUC Lactate: 0.59 (0.57-0.62)

AUC CRP: 0.58 (0.55-0.60)

**Supplementary Figure 4:** ROC curve for MAKE performance prediction including using Gal-3, pNGAL, Lactate at admission and C-reactive protein **(A)** and for AKI performance **(B)**.

*Gal-3: galectin 3, MAKE: Major adverse kidney event*, ROC: Receiver operating characteristic

| Variable | AUC CI_95%_ | Difference AUC CI_95%_ (logrank) | p-value | Continuous NRI CI_95%_ | p-value | IDI CI_95%_ | p-value |
| --- | --- | --- | --- | --- | --- | --- | --- |
| Screat | 0.74 (0.72-0.76) |  |  |  |  |  |  |
| Screat + Gal-3 | 0.77 (0.75-0.8) | 0.04 (0.02-0.05) | <0.001 | 0.35 (0.24-0.46) | <0.001 | 0.04 (0.02- 0.05) | <0.001 |
| SAPSII | 0.66 (0.64-0.69) |  |  |  |  |  |  |
| SAPSII+Gal-3 | 0.77 (0.75-0.79) | 0.11 (0.09-0.13) | <0.001 | 0.74 (0.64-0.85) | <0.001 | 0.17 (0.14-0.19) | <0.001 |
| pNGAL | 0.77 (0.74-0.79) |  |  |  |  |  |  |
| pNGAL+Gal-3 | 0.79 (0.77-0.81) | 0.02 (0.01-0.04) | <0.001 | 0.44 (0.33-0.55) | <0.001 | 0.06 (0.05-0.08) | <0.001 |
| Screat+SAPSII | 0.77 (0.75-0.79) |  |  |  |  |  |  |
| Screat+SAPSII+Gal-3 | 0.79 (0.77-0.81) | 0.02 (0.01-0.03) | <0.001 | 0.35 (0.24-0.46) | <0.001 | 0.03 (0.02-0.05) | <0.001 |
| Screat+SAPSII+pNGAL | 0.79 (0.77-0.81) |  |  |  |  |  |  |
| Screat+SAPSII+pNGAL+Gal-3 | 0.8 (0.78-0.82) | 0.01 (-0.01-0) | <0.001 | 0.27 (0.16-0.38) | <0.001 | 0.01 (0.01-0.03) | <0.001 |

**Supplementary Table 3**: Values of prediction performance and reclassification of models with or without Gal-3 for MAKE with AUC ROC, Continuous NRI and IDI.

*Gal-3: galectin 3, MAKE: Major adverse kidney event, SAPSII: Simplified acute physiology score, Screat: serum creatinine, NRI: Net reclassification importance, IDI: Integrated discrimination improvement.*

**C.**

**B.**

**A.**


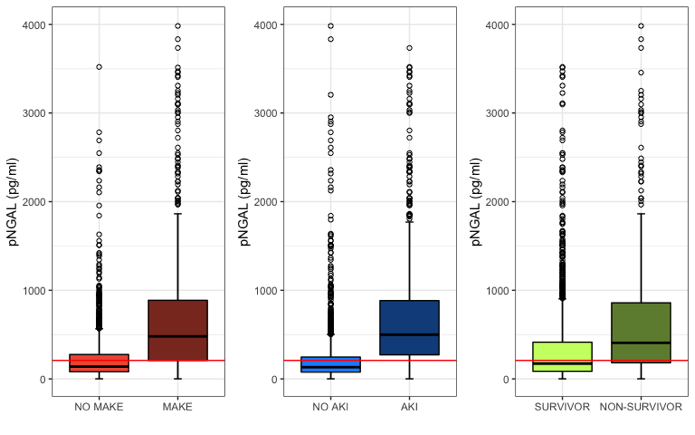


**E.**

**D.**


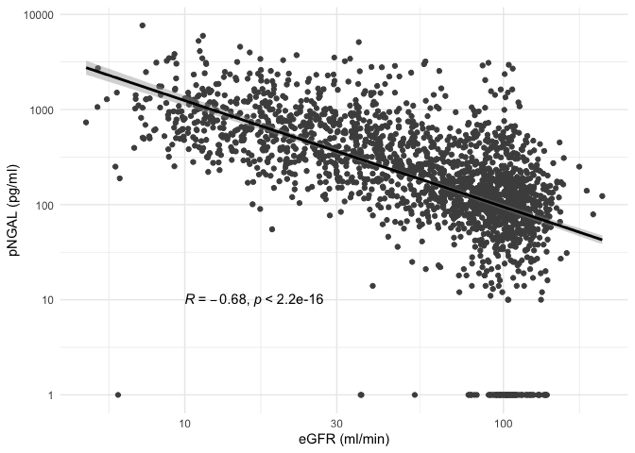

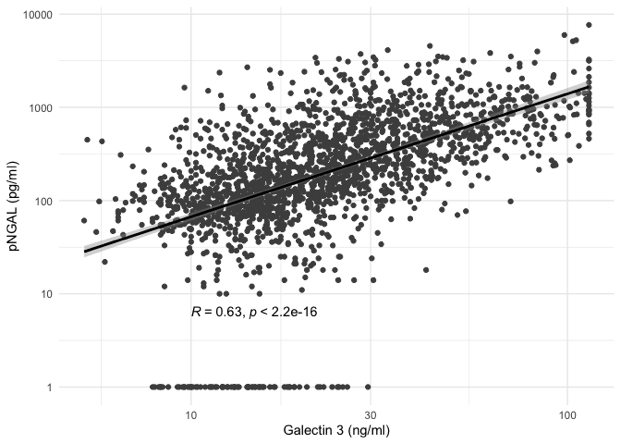


**Supplementary Figure 5:** NGAL level for MAKE and NO MAKE **(A)**, NGAL level in patient with AKI and without AKI **(B)**, NGAL level for survivor and non-survivor patients **(C)**. Correlation of NGAL level with eGFR expressed with a logarithmic scale **(D)**, Correlation of NGAL level with Gal-3 level **(E)**,

*Gal-3: galectin 3, MAKE: Major adverse kidney event, SAPSII: Simplified acute physiology score, Screat: serum creatinine, AKI: Acute kidney injury*, *CKD: Chronic kidney disease, pNGAL: plasma NGAL.*

*p-value: ns>0.05, *: 0.05-0.01, **: 0.01-0.001, *** < 0.001*


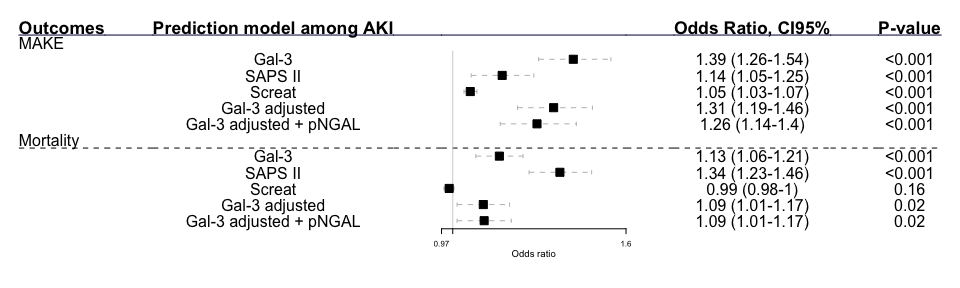


**A.**

**B.**

adm

adm


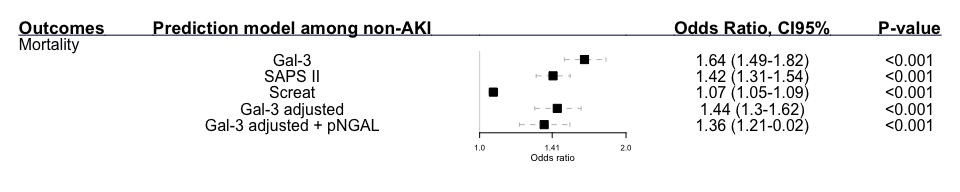


adm

**Supplementary Figure 6.** Association of Gal-3, SAPSII, Screat, Gal-3 adjusted and Gal-3 adjusted + pNGAL among patients with AKI for MAKE and Mortality **(A)**, association of Gal-3, SAPSII, Screat, Gal-3 adjusted and Gal-3 adjusted + pNGAL among patients with no AKI for MAKE and Mortality **(B**). Gal-3 association was adjusted with gender, age, CKD, vasopressor treatment, SAPS II, Charlson score, and lactate value at admission. For AKI association. OR for continuous variables (Gal-3, SAPSII, Screat_adm_) were standardized for each 10-unit change, for NGAL 100-unit change.

*Gal-3: galectin 3, MAKE: Major adverse kidney event, SAPSII: Simplified acute physiology score, Screat: serum creatinine, AKI: Acute kidney injury*, *CKD: Chronic kidney disease.*


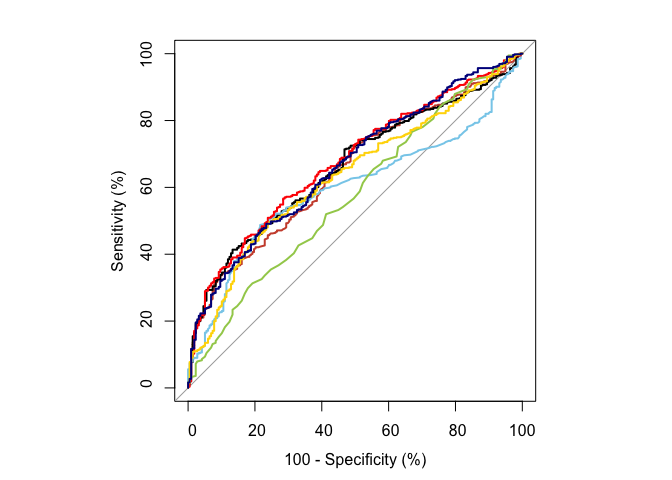


A.

**MAKE**

**among AKI**

**MORTALITY**

**among AKI**

Gal-3: 0.66 CI_95%_[0.62-0.70]

SCreat_adm_: 0.61 CI_95%_[0.57-0.65]

SAPS II: 0.57 CI_95%_[0.52-0.61]

Gal-3+Screat_adm_: 0.67 CI_95%_[0.63-0.71]

Gal-3+Screat_adm_+SAPSII: 0.68 CI_95%_[0.64-0.72]

Screat_adm_+SAPS II: 0.65 CI_95%_[0.61-0.69]

SAPS II+Gal-3: 0.67 CI_95%_[0.63-0.71]

*Gal-3 vs Screat_adm_, p-value = 0.05*

*Gal-3 vs SAPS2, p-value < 0.001*

*Gal-3 vs Gal-3+Screat_adm_, p-value=0.06*

*Gal-3 vs Gal-3+Screat_adm_,+SAPS II, p-value = 0.01*

*Gal-3 vs Screat_adm_+SAPSII, p-value=0.64*

*Gal-3 vs SAPSII+Gal-3, p-value = 0.11*


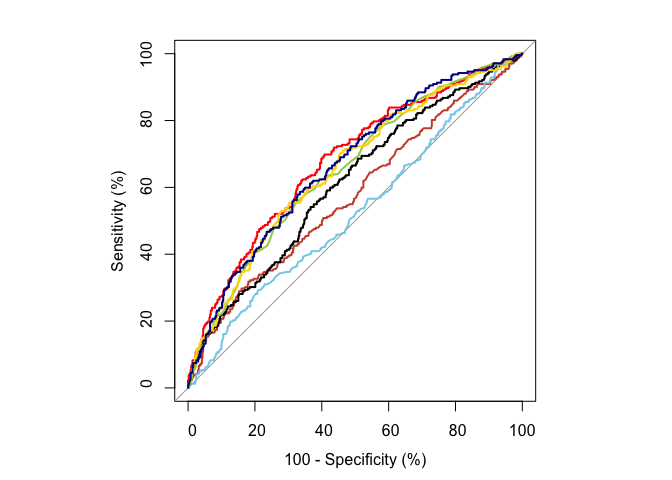


B.

Gal-3: 0.57 CI_95%_[0.53-0.62]

SCreat_adm_: 0.53 CI_95%_[0.48-0.57]

SAPS II: 0.65 CI_95%_[0.61-0.69]

Gal-3+Screat_adm_: 0.61 CI_95%_[0.57-0.65]

Gal-3+Screat_adm_+SAPS II: 0.68 CI_95%_[0.64-0.72]

Screat_adm_+SAPS II: 0.61 CI_95%_[0.57-0.65]

SAPS II+Gal-3: 0.67 CI_95%_[0.63-0.70]

*Gal-3 vs Screat_adm_, p-value=0.22*

*Gal-3 vs SAPS2, p-value=0.01*

*Gal-3 vs Gal-3+Screat_adm_, p-value=0.05*

*Gal-3 vs Gal-3 + Screat_adm_,+SAPS II p-value < 0.001*

*Gal-3 vs Screat_adm_+SAPSII, p-value=0.01*

*Gal-3 vs SAPSII+Gal-3, p-value < 0.001*

**Supplementary Figure 7:** ROC curve among AKI for MAKE **(A)** and mortality **(B)** performance prediction including using Gal-3, SAPSII and Screat_adm_ parameters.

*Gal-3: galectin 3, MAKE: Major adverse kidney event, SAPSII: Simplified acute physiology score, Screat_adm_*: Serum creatinine at admission, ROC: Receiver operating characteristics


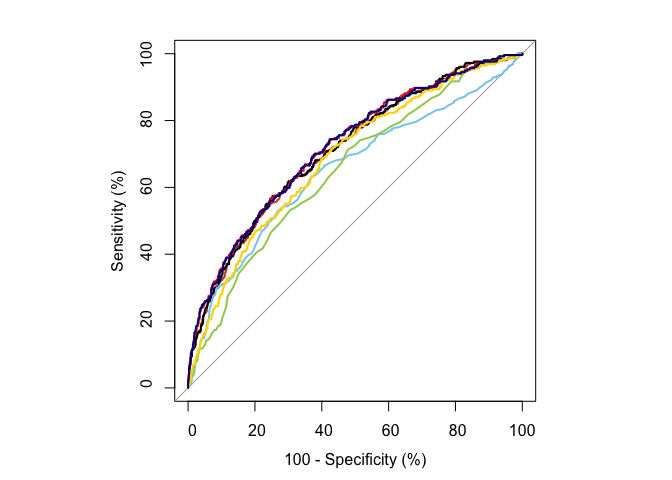


**MORTALITY**

**among no AKI**

Gal-3: 0.71 CI_95%_[0.67-0.74]

SCreat_adm_: 0.65 CI_95%_[0.61-0.69]

SAPS II: 0.65 CI_95%_[0.61-0.69]

Gal-3+Screat_adm_: 0.71 CI_95%_[0.68-0.75]

Gal-3+Screat_adm_+SAPS II: 0.72 CI_95%_[0.68-0.76]

Screat_adm_+SAPS II: 0.69 CI_95%_[0.65-0.72]

SAPS II+Gal-3: 0.72 CI_95%_[0.68-0.75]

*Gal-3 vs Screat_adm_, p-value=0.01*

*Gal-3 vs SAPS2, p-value=0.01*

*Gal-3 vs Gal-3+Screat_adm_, p-value=0.09*

*Gal-3 vs Gal-3 + Screat_adm_,+SAPS II p-value =0.37*

*Gal-3 vs Screat_adm_+SAPSII, p-value=0.25*

*Gal-3 vs SAPSII+Gal-3, p-value=0.4*

**Supplementary Figure 8:** ROC curve among no AKI patients for mortality performance prediction including using Gal-3, SAPSII and Screat_adm_ parameters.

*Gal-3: galectin 3, MAKE: Major adverse kidney event, SAPSII: Simplified acute physiology score, Screat_adm_*: Serum creatinine at admission, ROC: Receiver operating characteristic


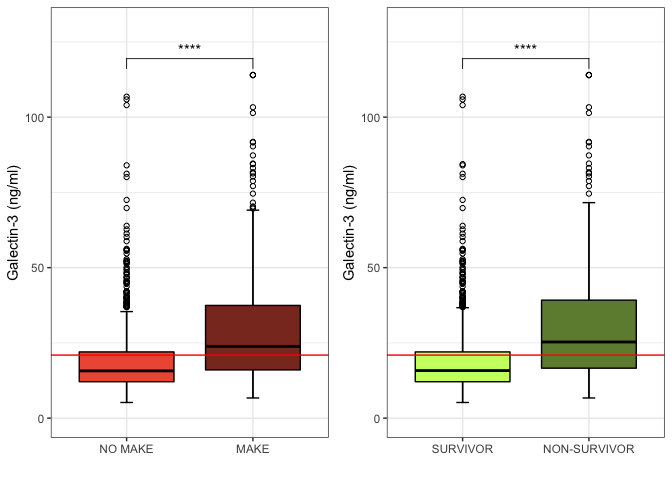


B.

Among AKI patients

A.

Among no-AKI patients


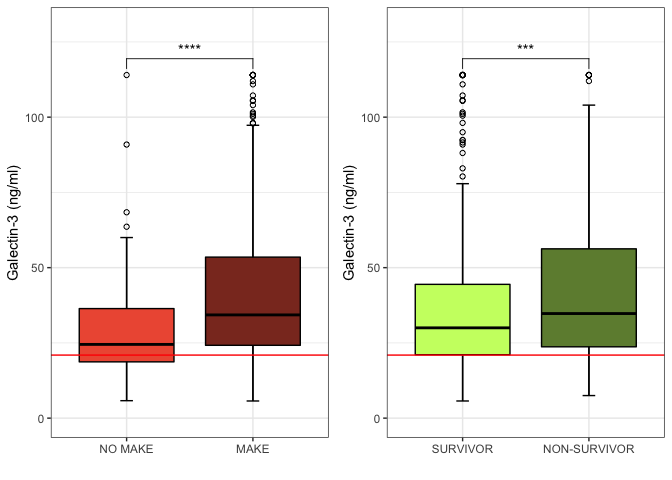


C.

**Supplementary figure 9:** Gal-3 level for MAKE and NO MAKE among patients with AKI **(A),** Gal-3 level for survivor and non-survivor patients among patients with AKI **(B)**, , Gal-3 level for survivor and non-survivor patients among patients with no-AKI **(C)**


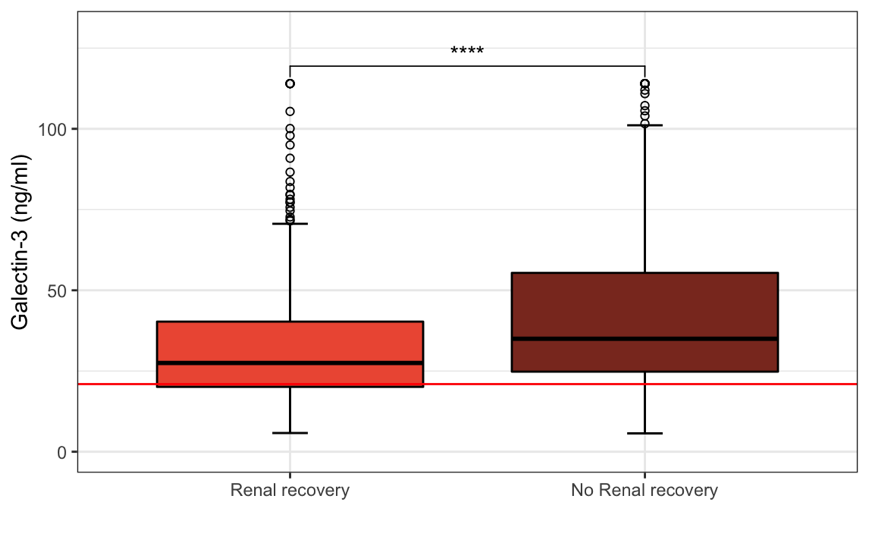


**Supplementary Figure 10:** Gal-3 level for patient with renal recovery and no renal recovery among patients with AKI.

|  | Renal recovery  (N=401) | | No renal recovery  (N=373) | P-value |
| --- | --- | --- | --- | --- |
| Age - years (median [Q1, Q2]) | 68 [57, 78] | 67 [56, 75] | | 0.019 |
| Female (%) | 127 (31.7) | 120 (32.2) | | 0.942 |
| BMI (median [Q1, Q2]) | 27.7 [24.1, 32.5] | 27.8 [24.4, 31.3] | | 0.899 |
| SOFA admission – score (median [Q1, Q2]) | 9 [6, 11] | 9 [6, 12] | | 0.555 |
| SAPS II admission – score (median [Q1, Q2]) | 56 [42, 70] | 57 [42, 69] | | 0.913 |
| **Admission diagnostic (%)** |  |  | | 0.643 |
| Other (%) | 54 (13.6) | 41 (11.2) | |  |
| Shock and cardiac arrest (%) | 71 (17.9) | 80 (21.9) | |  |
| Acute cardiac failure (%) | 32 (8.1) | 28 (7.7) | |  |
| Acute respiratory failure (%) | 63 (15.9) | 49 (13.4) | |  |
| Secondary to surgery (%) | 38 (9.6) | 35 (9.6) | |  |
| Severe sepsis (%) | 138 (34.8) | 133 (36.3) | |  |
| **Comorbidities** |  |  | |  |
| Chronic heart failure (%) | 41 (10.2) | 37 (9.9) | | 0.983 |
| Diabetes mellitus (%) | 87 (21.7) | 85 (22.8) | | 0.780 |
| Chronic hypertension (%) | 230 (57.4) | 188 (50.4) | | 0.062 |
| Chronic dyslipidemia (%) | 94 (23.4) | 82 (22) | | 0.691 |
| Chronic peripheral vascular disease (%) | 49 (12.2) | 46 (12.3) | | 1.000 |
| Chronic COPD (%) | 72 (18) | 53 (14.2) | | 0.188 |
| Chronic liver disease (%) | 31 (7.7) | 44 (11.8) | | 0.074 |
| Chronic renal disease (%) | 52 (13) | 71 (19) | | 0.027 |
| Chronic malignant tumor (%) | 52 (13) | 65 (17.4) | | 0.103 |
| Chronic inflammatory disease (%) | 14 (3.5) | 13 (3.5) | | 1.000 |
| **Chronic treatment** |  |  | |  |
| Aldosterone agonist (%) | 2 (0.5) | 2 (0.5) | | 1.000 |
| Diuretics (%) | 114 (28.6) | 100 (27.) | | 0.691 |
| ACE inhibitors or angiotensin II receptor blockers (%) | 150 (37.6) | 108 (29.2) | | 0.017 |
| **Physiological admission parameters** |  |  | |  |
| Systolic blood pressure – mmHg (median [Q1, Q2]) | 121.5 [107, 137] | 119.5 [106, 137] | | 0.698 |
| Diastolic blood pressure – mmHg (median [Q1, Q2]) | 59 [51, 67.8] | 58 [50, 68] | | 0.608 |
| Diuresis during the first 24 hours – ml (median [Q1, Q2]) | 1022.5 [580, 1800] | 790 [175, 1603.8] | | <0.001 |
| **Biological admission parameters** |  |  | |  |
| Admission plasmatic creatinine - µmol/l (median [Q1, Q2]) | 140 [113, 195] | 184 [112, 264] | | <0.001 |
| Admission plasma lactate dosage - mmol/l (median [Q1, Q2]) | 1.5 [1.1, 2.3] | 1.7 [1.1, 2.5] | | 0.036 |
| Galectin-3 admission dosage – ng/ml (median [Q1, Q2]) | 27.5 [20.1, 40.3] | 35 [24.8, 55.4] | | <0.001 |
| NGAL admission dosage – µg/l (median [Q1, Q2]) | 403 [212, 696.5] | 628.5 [348.2, 1064.8] | | <0.001 |
| **Renal Outcomes** |  |  | |  |
| MAKE (%) | 180 (44.9) | 373 (100) | | <0.001 |
| Renal function | 0 (0) | 0 (0) | |  |
| AKI (%) | 401 (100) | 373 (100) | |  |
| KDIGO 1 (%) | 195 (48.6) | 50 (13.4) | |  |
| KDIGO 2 (%) | 60 (15) | 59 (15.8) | |  |
| KDIGO 3 (%) | 146 (36.4) | 264 (70.8) | |  |
| **General outcomes** |  |  | |  |
| Mortality (%) | 100 (24.9) | 142 (38.1) | | <0.001 |
| In ICU length of stay – days (median [Q1, Q2]) | 14 [8, 23] | 15 [9, 28] | | 0.002 |
| Hospital Length of stay – days (median [Q1, Q2]) | 25 [15, 41] | 24 [13, 41] | | 0.546 |
| **Treatments during hospitalization** |  |  | |  |
| Vasopressors (%) | 358 (89.3) | 332 (89) | | 0.996 |
| Renal replacement therapy (%) | 109 (27.2) | 238 (63.8) | | <0.001 |
|  |  |  | |  |

**Supplementary Table 4**: Characteristics table of patient according to renal recovery among AKI patients.

*BMI: body mass index, SOFA: Sequential organ failure assessment, SAPSII: Simplified acute physiology score 2, COPD: Chronic obstructive pulmonary disease, ACE: Angiotensin-converting enzyme, AKI: Acute kidney injury, KDIGO: Kidney Disease: Improving Global Outcomes, ICU: intensive care unit.*


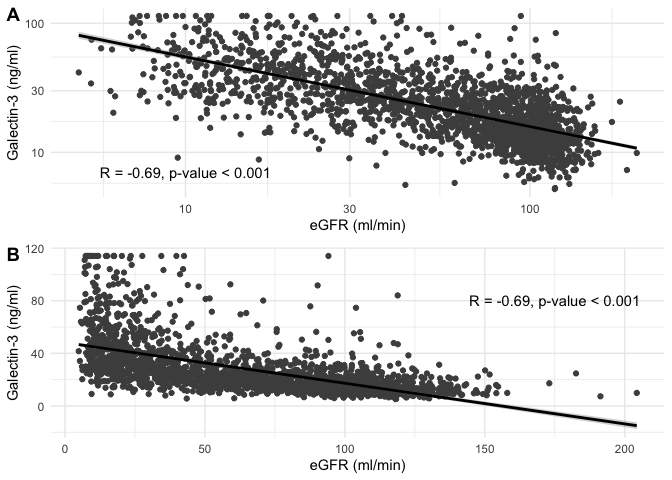


**Continuous scale**

**Log scale**


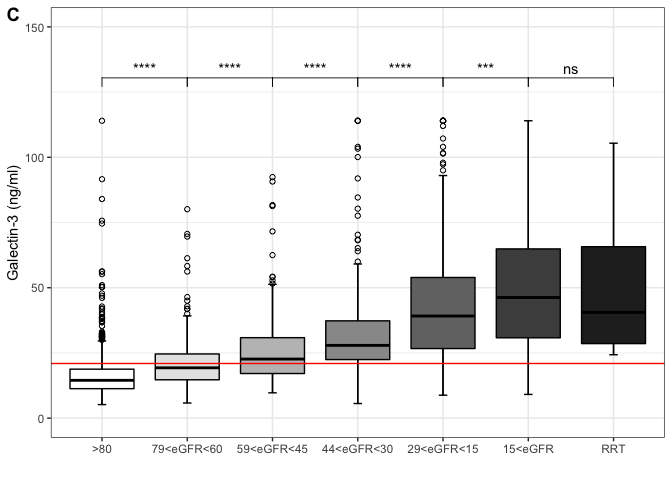


eGFR (ml/min)

Median Gal-3

**Supplementary Figure 11:** Correlation of Gal-3 level with eGFR expressed with a logarithmic scale **(A)**, with a continuous scale **(B)**, Gal-3 level with eGFR stratified severity subdivision **(C)**.

*eGFR: estimated glomerular filtration rate, RRT: Renal replacement therapy.*

*p-value: ns > 0.05, *<0.05-0.01, **<0.01-0.001, ***<0.001-0.0001, ****<0.0001.*


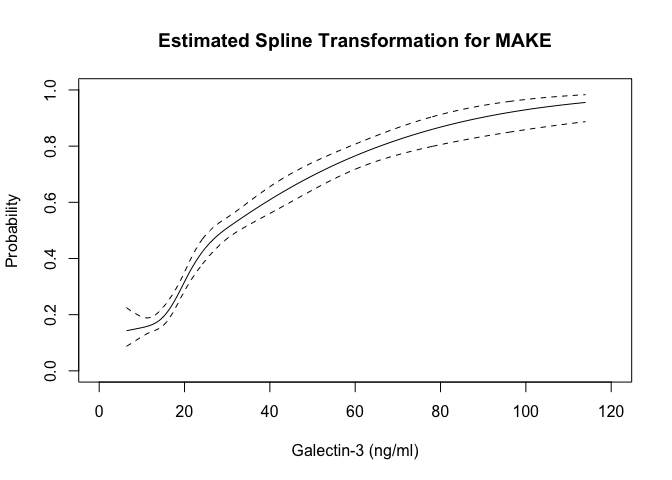


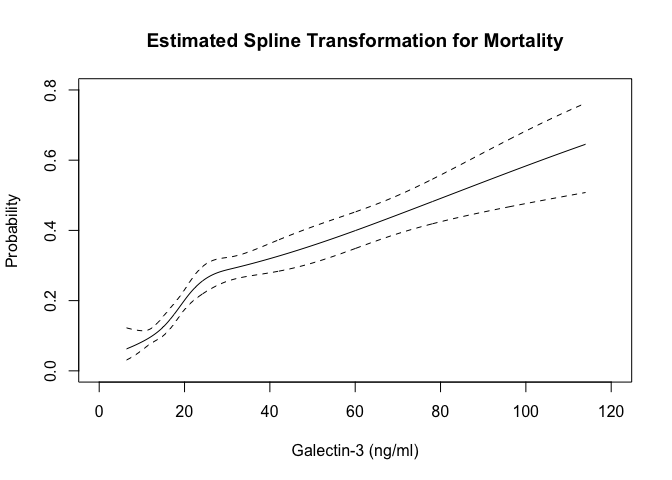


**B.**

**A.**

**Supplementary Figure 12:** Estimate spline transformation using logistic regression for Gal-3 association with MAKE **(A)**, Mortality **(B)**.

*MAKE: Major adverse kidney event, AKI: Acute kidney injury*

*
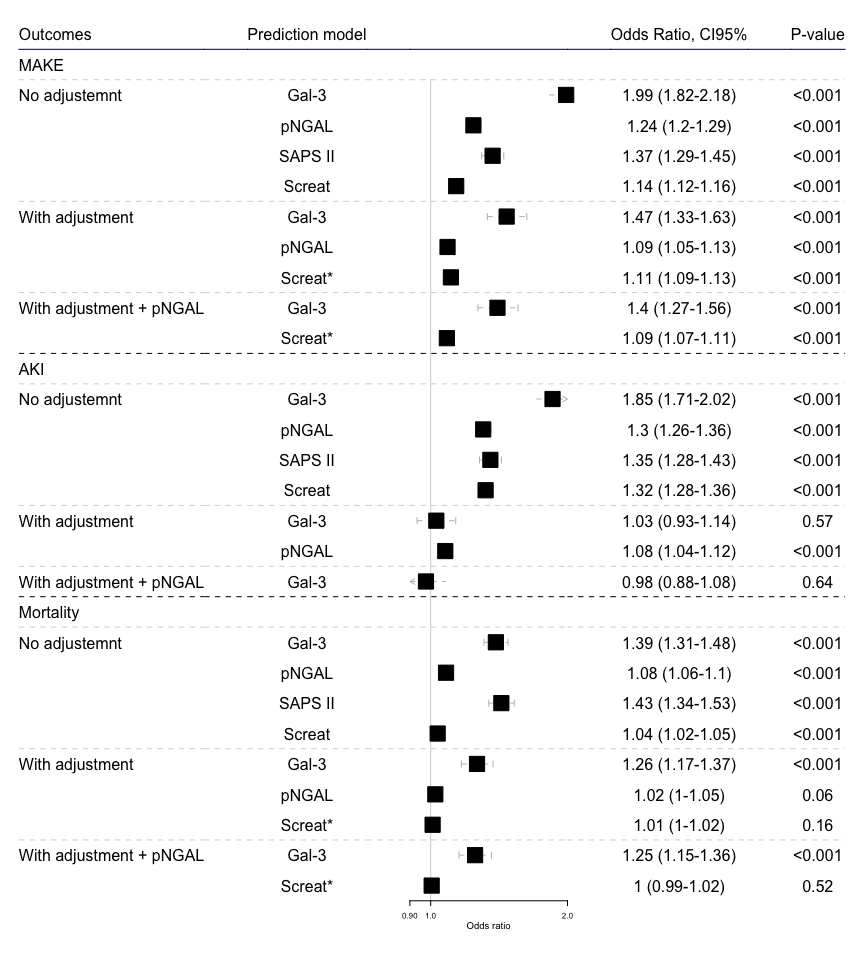
*

adm

adm

adm

adm

adm

adm

adm

**Supplementary Figure 13:** Gal-3, SAPSII, Screat_adm_, pNGAL, Gal-3 after adjustment and Screat_adm_ after adjustment, Gal-3 after adjustment including pNGAL and Screat_adm_ after adjustment including pNGAL association with MAKE, AKI and Mortality **after exclusion of CKD and RRT patients**. Gal-3 association was adjusted with gender, age, CKD, vasopressor treatment, SAPS II, Charlson score, Screatadm and lactate value at admission. For AKI association, Screat was not include in the adjustment model. OR for continuous variables (Gal-3, SAPSII, Screatadm) were standardized for each 10-unit change and pNGAL for each 10-fold unit change.

*These model do not include Screat_adm_

OR: odds ratio, CI: confidence interval, SAPS II: Simplified acute physiology score II, MAKE: Major adverse kidney event, Gal-3: galectin 3, Screat: serum creatinine, CKD: Chronic kidney disease
